# Annotation and classification of the bovine T cell receptor delta genes

**DOI:** 10.1186/1471-2164-11-100

**Published:** 2010-02-09

**Authors:** Carolyn TA Herzig, Marie-Paule Lefranc, Cynthia L Baldwin

**Affiliations:** 1 Department of Veterinary and Animal Sciences, University of Massachusetts, Amherst, MA 01003, USA; 2 IMGT, Institut Universitaire de France, Laboratoire d'Immuno Génétique Moléculaire, Université Montpellier 2, UPR CNRS 1142, Montpellier, France; 3 Program in Molecular and Cellular Biology, University of Massachusetts, Amherst, MA 01003, USA; 4 Current address: Department of Epidemiology, Columbia University, New York, NY 10032, USA

## Abstract

**Background:**

γδ T cells differ from αβ T cells with regard to the types of antigen with which their T cell receptors interact; γδ T cell antigens are not necessarily peptides nor are they presented on MHC. Cattle are considered a "γδ T cell high" species indicating they have an increased proportion of γδ T cells in circulation relative to that in "γδ T cell low" species such as humans and mice. Prior to the onset of the studies described here, there was limited information regarding the genes that code for the T cell receptor delta chains of this γδ T cell high species.

**Results:**

By annotating the bovine (*Bos taurus*) genome Btau_3.1 assembly the presence of 56 distinct T cell receptor delta (TRD) variable (V) genes were found, 52 of which belong to the TRDV1 subgroup and were co-mingled with the T cell receptor alpha variable (TRAV) genes. In addition, two genes belonging to the TRDV2 subgroup and single TRDV3 and TRDV4 genes were found. We confirmed the presence of five diversity (D) genes, three junctional (J) genes and a single constant (C) gene and describe the organization of the TRD locus. The TRDV4 gene is found downstream of the C gene and in an inverted orientation of transcription, consistent with its orthologs in humans and mice. cDNA evidence was assessed to validate expression of the variable genes and showed that one to five D genes could be incorporated into a single transcript. Finally, we grouped the bovine and ovine TRDV1 genes into sets based on their relatedness.

**Conclusions:**

The bovine genome contains a large and diverse repertoire of TRD genes when compared to the genomes of "γδ T cell low" species. This suggests that in cattle γδ T cells play a more important role in immune function since they would be predicted to bind a greater variety of antigens.

## Background

T lymphocytes can be subdivided into at least two types based on the expression of either the αβ or γδ T cell receptor. Although both perceive antigen, they differ in the types of antigens with which they react. αβ T cell receptors react with antigenic protein peptides in the context of self major histocompatibility complex (MHC) proteins while γδ T cell receptors may react with proteins but this does not involve MHC presentation. They also react with autologous molecules on cells [1-6] as well as nonproteinaceous molecules [7]. The gene repertoires that code for the γδ T cell receptor chains and the T cell receptor gamma (TRG) and delta (TRD) gene locus organizations have been extensively described for humans and mice but to a lesser extent for the artiodactyls which includes ruminants and swine. These latter are "γδ T cell high" species because of their high levels of γδ T cells in circulation ("γδ T cell low" species exhibit much lower levels of γδ T cells in circulation). It is clear that while both αβ and γδ T cells have large and diverse T cell receptor gene repertoires [8-14], "γδ T cell high" species have a TRD gene repertoire that is much more extensive than that in the "γδ T cell low" species [15-21]. The bovine (*Bos taurus*) locus organization and TRD gene repertoire are the subject of this work.

T cell receptor delta and beta chains are encoded by the rearrangement of variable (V), joining (J) and diversity (D) genes making them more complex than the T cell receptor gamma and alpha chains which lack D gene products. In all mammals evaluated the genes encoding the T cell receptor beta and gamma chains are found at the T cell receptor beta (TRB) and TRG loci, respectively. The genes that encode the T cell receptor delta and alpha chains are found at a single chromosomal location with the TRD genes embedded within the T cell receptor alpha (TRA) locus. For both humans and mice these are located on chromosome 14 and encompass over 1 megabase (Mb) for the combined TRA/TRD locus [13]. The TRD locus embedded within the TRA locus spans approximately 60 kb in humans and 275 kb in mice. The loci comprise TRDV genes (two in humans and five in mice), followed by TRDD genes (three in humans and two in mice), TRDJ genes (four in humans and two in mice), a single TRDC gene and an additional TRDV gene that is located 3' of the TRDC gene in an inverted transcriptional orientation [10,14]. In addition, five and ten functional TRAV/DV genes (that rearrange to either TRDD or TRAJ) have been identified for humans and mice, respectively, and these are found upstream of the embedded TRD locus [10,14].

Limited evidence derived from analysis of cDNA clones from cattle, sheep and swine, as well as limited germline information for sheep, suggests that the general organization of the bovine TRA/TRD locus does not differ greatly from that of humans and mice [17,20]. It also suggests that a much larger repertoire of TRDV genes exists for cattle, as well as for the other "γδ T cell high" species sheep and swine [15,17,20,21]. Indeed, recent mapping of the bovine TRA/TRD locus identified over 100 TRDV and over 300 TRAV or TRAV/DV genes in cattle [22]. For cattle, four genes belonging to three different small subgroups (TRDV2, TRDV3 and TRDV4) have been identified that are orthologous to those same subgroups in sheep [17,23]. In contrast, while the bovine TRDV1 genes have been found to be related to the single TRDV1 gene that occurs in humans, expansion of the TRDV1 subgroup accounts for the larger number of TRDV genes in the "γδ T cell high" species [15,17,20-22]. The swine and sheep TRDV1 subgroups have been estimated to contain at least 31 and 40 genes, respectively [15,21] while the bovine TRDV1 subgroup has been reported to contain at least 37 genes [20]. However, current knowledge about ruminant and swine TRD genes is predominantly based on cDNA evidence rather than on genomic DNA. Because of this, it has been unclear whether the large number of cDNA TRDV1 sequences is the result of multiple genes or polymorphisms among animals; thus, there has been no clear way to classify and name these transcripts. By annotating the bovine genome Btau_3.1 assembly, here we demonstrated the presence of 56 TRDV genes, 52 of which belong to the TRDV1 subgroup. We also proposed TRDV1 sets to classify the bovine and ovine TRDV1 genes based on their phylogenetic grouping.

The existence of multiple D and J genes also contributes to diversity of possible T cell receptor delta chain amino acid sequences since a particular TRDV gene is expected to be able to recombine with any D and J gene. The region of the protein derived from the V-D-J gene rearrangement is known as the complementarity determining region 3 (CDR3), while the CDR1 and CDR2 loops are germline-encoded by the TRDV gene. The V-D-J gene rearrangement involves recognition of the recombination signal (RS) sequences for subsequent DNA cleavage by the enzymes RAG1 and RAG2 [24]. T cell receptor delta chain sequence diversity is augmented by junctional flexibility and the addition of N and P nucleotides. RS flank the V, D and J genes and are composed of highly conserved heptamer and nonamer sequences separated by either a 12 base pair (bp) spacer (located 5' of TRDD and TRDJ genes) or a 23 bp spacer (located 3' of TRDV and TRDD genes). Since the conserved heptamer and nonamer sequences and spacer lengths have been found to be important for efficient recombination [25] they were also evaluated here. Finally, recent evidence demonstrated that diversity of T cell receptor delta chain sequences is amplified by the occurrence of multiple TRDD genes within a single CDR3 [20,26]. Thus we also evaluated TRDD gene usage by analyzing cDNA sequences and report those findings here.

## Results

### Gene structure and genomic organization of bovine TRD genes

Here we set out to annotate and classify the bovine TRD genes. Prior to the initiation of the studies described here many cDNA sequences representing TRDV1 gene transcripts had been reported [16-20,27] but the actual number of functional bovine TRDV1 genes and their sequences was unknown. As a result it was not possible to differentiate between cDNA sequences derived from distinct genes and those that represented polymorphisms of the same gene among animals. Furthermore, the existence of TRDV2, TRDV3 and TRDV4 genes had been demonstrated [17], along with the TRDJ and TRDD genes [20], but their numbers and placement within the bovine genome had not been definitively described.

**Figure 1:**
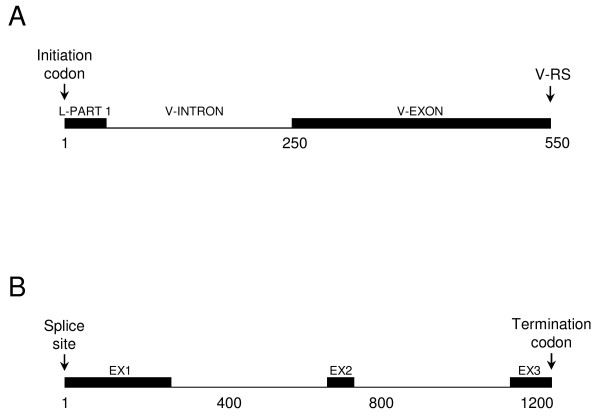
**Schematic representation of TRD gene exon-intron structures**. (A) Representative structures of TRDV genes (based on genomic sequence for TRDV1au, GLEAN_15708), which contain two exons, and (B) the single TRDC gene (GLEAN_19705), which contains three exons, are shown. Scale is shown in base pair increments beneath the schematic. V-RS, recombination signal sequence of a V gene.

The exon-intron structures of TRDV genes (Figure 1A) and of the single TRDC gene (Figure 1B) were determined. A TRDV gene encompasses a coding region of approximately 560 bp and comprises two exons. The first exon, L-PART1, is 49, 55 and 37 bp in length for TRDV1/TRDV2, TRDV3 and TRDV4, respectively. The second exon, V-EXON, is between 271 and 335 bp in length for TRDV1, and is 299, 293 and 309 bp long for TRDV2, TRDV3 and TRDV4, respectively. The TRDC gene encompasses a coding region of 1282 bp comprising three exons. RS sequences were identified, as expected, adjacent to the 3' end of each V-EXON (Figure 1A) while the termination codon was at the 3' end of TRDC exon 3.

Table 1 summarizes the proposed functional (or Open Reading Frame [ORF]) TRD genes that were identified within the bovine Btau_3.1 assembly. Most genes were identified within regions that were not placed on a chromosome (i.e. were found on ChrUn). However, it is expected that, like in primates and rodents, these genes are all located within the TRA/TRD locus which in cattle is on chromosome 10. The bovine consensus gene set (known as GLEAN) was used to identify gene prediction models of TRD genes. Where applicable, GLEAN numbers are listed in Table 1, along with the Bovine Genome Scaffold identification number, location and orientation. It should be noted that the orientation of each contig individually, as well as in relation to other contigs, is not definitive at this point for the bovine Btau_3.1 assembly. All putatively functional (or ORF) genes (as determined based on criteria described in Methods) are shown. Based on analysis of flanking and intronic genomic sequence it was determined that many of these gene models might represent the same genes although they were found more than once. It is possible that this is due to an anomaly in the Btau_3.1 assembly, although it cannot be ruled out that those gene models do indeed represent duplicated genes that are present in the genome. 67 TRDV1, three TRDV2, two TRDV3 and one TRDV4 genes were identified; after consideration of multiple gene models that might be representative of a single gene we propose the presence of 52 TRDV1, two TRDV2, one TRDV3 and one TRDV4 genes. The number of TRDV2, TRDV3 and TRDV4 genes is fairly consistent with what has been previously reported [17,20], although evidence for two TRDV3 genes has been presented by others [20]. In addition to the TRDV genes, five TRDD genes, three TRDJ genes and a single TRDC gene were identified, also consistent with previous reports [17,20].

**Table 1:** Annotated TRD genes

Gene designation^1^	GLEAN number^2^	Scaffold Identification Number	Start	End	Orientation within the scaffold
TRDV1a	GLEAN_22158	ChrUn.139	314906	315465	+
TRDV1b	GLEAN_22157	ChrUn.139	303549	304112	+
TRDV1c^§§§§§^	GLEAN_22161	ChrUn.139	356577	357146	+
TRDV1d*	GLEAN_22150	ChrUn.139	75036	75601	-
TRDV1e*	GLEAN_22151	ChrUn.139	16460	17025	+
TRDV1f	GLEAN_22162	ChrUn.139	373301	373837	+
TRDV1g	GLEAN_17632	ChrUn.298	28570	29136	+
TRDV1h	GLEAN_17646	ChrUn.298	223460	224017	+
TRDV1i	GLEAN_17645	ChrUn.298	206764	207325	+
TRDV1j	GLEAN_17648	ChrUn.298	263421	263882	+
TRDV1k	GLEAN_02242	ChrUn.1758	21943	22497	-
TRDV1l	GLEAN_02239	ChrUn.1758	58028	58581	-
TRDV1m	GLEAN_18280	ChrUn.221	6077	6638	-
TRDV1n	GLEAN_18279	ChrUn.221	30283	30837	-
TRDV1o****	GLEAN_18269	ChrUn.221	174860	175436	-
TRDV1p	NA	ChrUn.221	220755	221325	-
TRDV1q^§§§^	GLEAN_18266	ChrUn.221	240734	241302	-
TRDV1r	GLEAN_18263	ChrUn.221	264437	265003	-
TRDV1s	GLEAN_05951	ChrUn.41	188111	188668	-
TRDV1t**	GLEAN_05971	ChrUn.41	313360	313923	+
TRDV1u	GLEAN_05970	ChrUn.41	294987	295550	+
TRDV1v***	GLEAN_05970	ChrUn.41	282485	282946	+
TRDV1w	GLEAN_05969	ChrUn.41	272858	273319	+
TRDV1x	GLEAN_05952	ChrUn.41	161524	162099	-
TRDV1y**	GLEAN_05991	ChrUn.41	661894	662457	+
TRDV1z	GLEAN_05990	ChrUn.41	642343	642906	+
TRDV1aa	GLEAN_05984	ChrUn.41	480039	480605	+
TRDV1ab***	GLEAN_05989	ChrUn.41	629990	630287	+
TRDV1ac	GLEAN_16627	ChrUn.158	178923	179480	-
TRDV1ad	GLEAN_16623	ChrUn.158	237111	237679	-
TRDV1ae	GLEAN_16625	ChrUn.158	209540	210077	-
TRDV1af	GLEAN_03708	ChrUn.129	414487	415056	+
TRDV1ag	GLEAN_03704	ChrUn.129	331405	331977	+
TRDV1ah	GLEAN_03700	ChrUn.129	300288	300855	+
TRDV1ai****	GLEAN_03707	ChrUn.129	390952	391528	+
TRDV1aj	GLEAN_03683	ChrUn.129	5640	6395	+
TRDV1al^◇^	GLEAN_10351	ChrUn.857	4172	4726	+
TRDV1am	GLEAN_10353	ChrUn.857	19494	20055	+
TRDV1an^§^	GLEAN_10354	ChrUn.857	28383	28939	+
TRDV1ao^§§^	GLEAN_10356	ChrUn.857	50213	50768	+
TRDV1ap	GLEAN_10558	ChrUn.857	74024	74592	+
TRDV1aq	GLEAN_15702	Chr10.28	20379	20949	-
TRDV1ar^◇◇◇◇◇^	GLEAN_15705	Chr10.28	58187	58723	+
TRDV1as	GLEAN_15706	Chr10.28	84670	85131	+
TRDV1at^◇^	GLEAN_15706	Chr10.28	97187	97750	+
TRDV1au^§^	GLEAN_15708	Chr10.28	115470	116026	+
TRDV1av^§§^	GLEAN_15709	Chr10.28	132846	133401	+
TRDV1aw^§§§§^	GLEAN_15699	Chr10.28	198285	198854	-
TRDV1ax	GLEAN_15696	Chr10.28	245707	246273	-
TRDV1ay^§§§^	GLEAN_19588	ChrUn.2578	31691	32259	-
TRDV1az	GLEAN_06130	ChrUn.1227	79925	80480	-
TRDV1ba	GLEAN_06133	ChrUn.1227	39695	40263	-
TRDV1bb	GLEAN_06131	ChrUn.1227	62992	63563	-
TRDV1bc^◇◇◇^	GLEAN_26185	ChrUn.2188	10476	11031	+
TRDV1bd	GLEAN_26186	ChrUn.2188	33687	34255	+
TRDV1be^§§§§^	GLEAN_21863	ChrUn.5529	9610	10170	-
TRDV1bf^§§§§§^	GLEAN_20556	ChrUn.4913	10675	11244	-
TRDV1bg	GLEAN_05967	ChrUn.41	250250	250849	+
TRDV1bh	GLEAN_04691	ChrUn.3566	5067	5528	+
TRDV1bi^◇^	GLEAN_04693	ChrUn.3566	19054	19618	+
TRDV1bj^◇◇^	GLEAN_10738	ChrUn.3620	538	1129	+
TRDV1bk^◇◇^	GLEAN_12353	ChrUn.3793	3915	4506	+
TRDV1bl^◇◇◇^	GLEAN_22424	ChrUn.3970	9390	9945	+
TRDV1bm	GLEAN_20555	ChrUn.10149	1843	2409	-
TRDV1bn^◇◇◇◇^	GLEAN_08205	ChrUn.3458	16972	17538	-
TRDV1bo^◇◇◇◇^	GLEAN_19099	ChrUn.2982	15173	15739	+
TRDV1bp^◇◇◇◇◇^	GLEAN_05881	ChrUn.4123	14961	15497	+
TRDV2-1	NA	ChrUn.158	345140	345657	-
TRDV2-2^¤^	GLEAN_10313	ChrUn.1907	43992	44509	+
TRDV2-2b^¤^	GLEAN_25296	ChrUn.1948	11302	11819	+
TRDV3-1^¤¤^	NA	ChrUn.7503	3142	3667	-
TRDV3-1b^¤¤^	GLEAN_19095	ChrUn.1002	77577	78102	+
TRDV4	GLEAN_19724	Chr10.30	396663	397245	+
TRDD1	NA	Chr10.30	504600	504612	-
TRDD2	NA	Chr10.30	488743	488757	-
TRDD3	NA	Chr10.30	449660	449672	-
TRDD4	NA	Chr10.30	441241	441249	-
TRDD5	NA	Chr10.30	417750	417760	-
TRDJ1	NA	Chr10.30	416717	416770	-
TRDJ2	NA	Chr10.30	406656	406598	-
TRDJ3	NA	Chr10.30	410369	410417	-
TRDC	GLEAN_19705	Chr10.30	402706	403988	-

^1^Gene designations with the same symbols denote gene models that are thought to be representative of the same gene based on analysis of genomic sequence.

^2^NA denotes identified genes lacking glean models.

**Figure 2:**
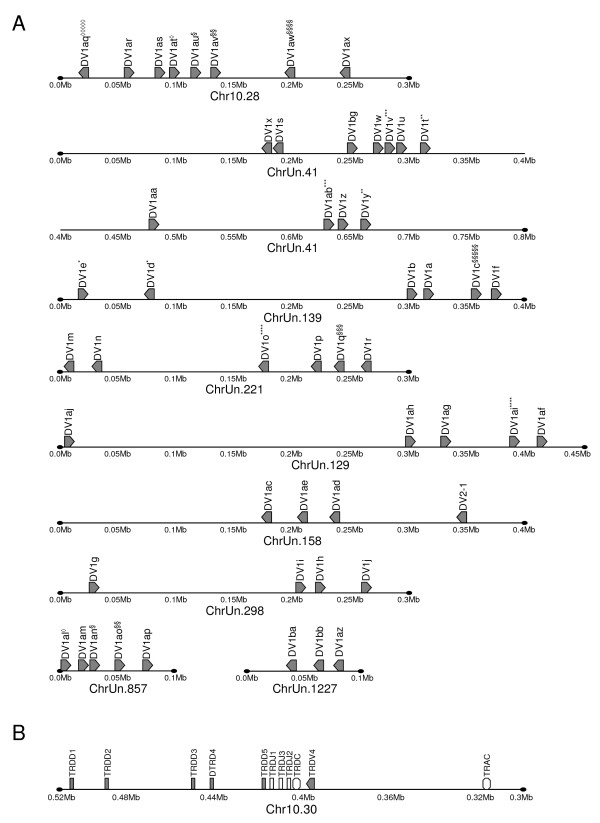
**Schematic representation of the bovine (*Bos taurus*) TRD locus organization**. (A) 52 unique TRDV1 genes were identified and are all predicted to be functional (or ORF) and found on chromosome 10, although many of them were unplaced in the Btau_3.1 assembly. Multiple TRDV genes were identified in two or three gene prediction models, as indicated, and labels correspond to those used in Table 1. However, evidence suggests that in each case only a single gene is represented. Genomic organization is shown in those cases when three or more TRDV genes were found within a single scaffolded region. TRA genes are not shown although in many cases occur among the TRDV genes. In addition to some TRDV1 genes, the schematic includes TRDV2-1 on ChrUn.158 (no other TRDV genes are included in the schematic). (B) Genomic organization of the five TRDD genes, 3 TRDJ genes and the single TRDC gene is shown, along with TRDV4 and TRAC. Gene designations, orientations and Bovine Genome Scaffold identifications are as indicated. The determination of TRDV gene orientation was based on the assembly of the Bovine Genome Scaffolds, it is possible that some scaffolds were assembled in an incorrect orientation individually and/or in relation to other scaffolds. Diagrams are shown to scale with base pair increments beneath the schematics. With the exception of TRAC, TRA genes are not shown. TRDV2-2 and TRDV3 are not shown because those genes were identified on scaffolds lacking additional TRDV genes.

The number of functional TRD genes among mammalian species is compared in Table 2. All species evaluated have genes for at least three TRDV subgroups. It is notable that humans have only a single TRDV1 gene (the mouse genes most closely related to human TRDV1 are named TRDV2-1 and TRDV2-2) while the TRDV1 subgroup in cattle and other artiodactyls are large, multigene families. Because of the disparity in numbers of TRDV1 genes in the human and cattle genomes, organization of their TRA/TRD loci cannot be compared. Furthermore, additional assembly of the bovine genome is required in order to definitively determine the size of the TRA/TRD locus, to refine the gene organization, and to evaluate duplication events that resulted in the large TRDV1 subgroup. Schematic representations for regions in which three or more TRDV genes were identified are shown (Figure 2A). Schematics are shown to scale and gene orientation is as indicated, based on the Btau_3.1 assembly. As in other species, a single TRDC gene (see Figure 2B) as well as one TRDV gene located downstream of TRDC in an inverted transcriptional orientation (named TRDV4 in cattle) were identified [17]. In some cases multiple gene models were found to represent the same gene (see Table 1, as indicated above, and Figure 2) and therefore subsequent analyses were performed after removing redundant sequences. It is likely that the occurrence of redundant sequences is a result of assembly anomalies; however, it is also possible that these duplicated TRDV genes are in fact present within the bovine genome.

**Table 2:** Number of TRD genes among species

Locus	TRD					
	**TRDV**					
					
**Group**	**TRDV1**	**TRDV2 to TRDV5**	**TRDD**	**TRDJ**	**TRDC**	**Reference**

Human	1	2	3	4	1	IMGT http://www.imgt.org and [14]
Mouse	1	5	2	2	1	IMGT http://www.imgt.org and [10]
Bovine	52	4	5	3	1	Described here
Ovine	> 40	3	?	4	1	[15,23]
Swine	> 31	6	?	4	1	[21]

### TRDV gene analysis

**Figure 3:**
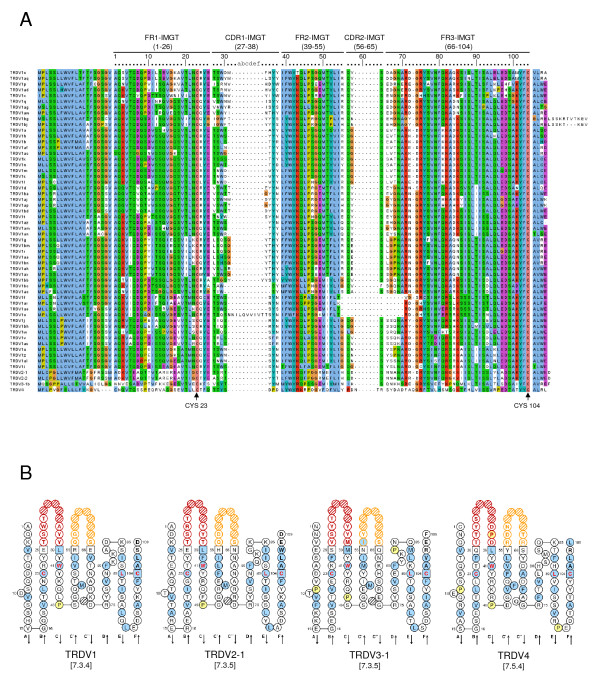
**TRDV gene sequences**. (A) TRDV deduced amino acid sequences were aligned with ClustalW2 using the default parameters and visualized with JalView. Analysis included all non-redundant genomic sequences (see Table 1 for GLEAN identification numbers). IMGT unique numbering for V-DOMAIN [29,32] is indicated above the alignment and conserved cysteines are indicated below the alignment. (B) IMGT Collier de Perles [33] are shown for TRDV1 (based on TRDV1a, GLEAN_22158), TRDV2-1 (no glean identification), TRDV3-1 (no glean identification) and TRDV4 (GLEAN_19724) and were determined using IMGT/V-QUEST [32]. The CDR-IMGT lengths are indicated.

Deduced amino acid sequences of the 56 TRDV genes identified here were initially aligned using ClustalW2 [28]. Alignments were refined in BioEdit according to the IMGT unique numbering for the V-DOMAIN [29], visualized with Jalview [30] and are shown (Figure 3A) with IMGT numbering and framework regions (FR) and CDR, as indicated. TRDV1 genes reported here are temporarily designated TRDV1a - TRDV1bp until the entire chromosomal sequence has been assembled. TRDV1 genes encode CDR1 that range in length from five to ten amino acids (except for TRDV1o which encodes an 18 amino acid long CDR1) while TRDV2, TRDV3 and TRDV4 genes all encode CDR1 that are seven amino acids in length. In contrast, most CDR2 of TRDV1, TRDV2 and TRDV3 genes were three amino acids in length, except for four of the TRDV1 genes as noted below, while that of TRDV4 was five amino acids long. In all but two cases (TRDV1bc and TRDV1w), TRDV1 and TRDV2 genes encode a QXS motif in the CDR2. Interestingly, TRDV1f, TRDV1ae, TRDV1ar and TRDV1o (see Figure 3A) all contained a deletion which spans the last FR2 amino acid to the fifth FR3 amino acid, resulting in a lack of CDR2 in those genes. An identical deletion had also been found in the sheep TRDV1S29 gene [15]. Also, the RS of TRDV1bg and TRDV1bj were found to occur farther downstream than those of the other TRDV1 genes identified here, resulting in a putative transcript that extends 15 (for TRDV1bg) or 12 (for TRDV1bj) amino acids beyond the conserved 2nd-CYS 104, as opposed to extending only three or four amino acids which is the case for the other TRDV1 genes. Because of the unusual nature of those six genes, until their expression by γδ T cells is validated they will be considered ORF instead of functional as defined by IMGT [31]. 2D structure graphical representations of TRDV1, TRDV2, TRDV3 and TRDV4 genes (Figure 3B) were generated using IMGT/V-QUEST [32] and the IMGT/Collier-de-Perles tool program [33]. The CDR1 and CDR2 loops are indicated in red and yellow, respectively. The amino acids 1st-CYS 23, CONSERVED-TRP 41, hydrophobic amino acid (Leu) 89 and 2nd-CYS 104, which are conserved in all four TRDV subgroups, are shown with letters in red.

**Figure 4:**
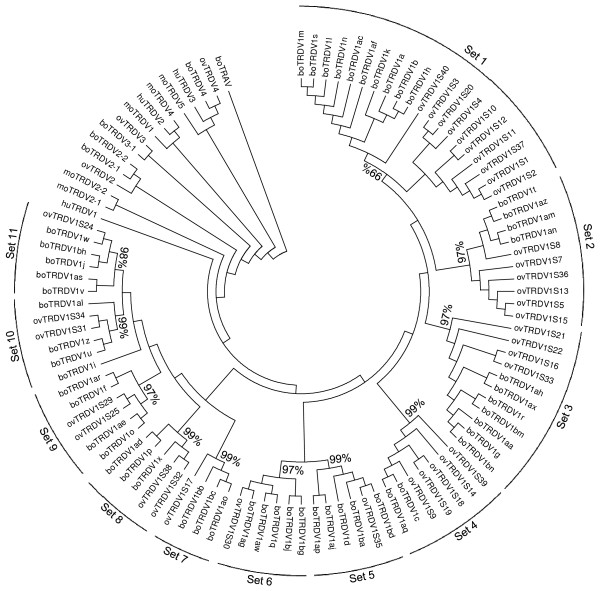
**Phylogenetic tree of bovine TRDV genes**. The Neighbor-Joining method [47] was used to classify TRDV genes using non-redundant bovine (bo) genomic TRDV sequences identified here and the previously classified ovine (ov), murine (mo) and human (hu) TRDV sequences (described in Methods). Bovine TRAV (GenBank accession number BC148926) was included in the analysis and was used to root the tree. The optimal tree with the sum of branch length = 5.37540770 is shown. Complete deletion to eliminate gaps was performed and the final dataset included a total of 207 positions. Eleven phylogenetic sets are indicated along with the percentage interior branch test value based on 1000 replicates for each set.

As for sheep, genes in the bovine TRDV1 family share many characteristics including a 20 amino acid long highly conserved leader region and the YFC motif found in the 3' end of FR3 (see Figure 3A). However, bovine TRDV1 genes described here were further classified into eleven sets by constructing a phylogenetic tree using the neighbor-joining method allowing us to compare them to equivalent human, mouse and ovine sequences (Figure 4). The grouping of ovine TRDV1 genes by this method corresponded with what had been previously described [15]. The tree shows grouping of bovine TRDV1 gene sequences into eleven sets, as indicated, and the percentage interior test values (based on 1000 replicates) are shown for each set. Classification was further supported based on CDR characteristics used to classify ovine TRDV1 genes [15] (Table 3). Previously described characteristics used to classify TRDV1 genes included CDR1 length (five, seven, nine or ten amino acids), the chemical characteristics of the amino acid at position 57 (within CDR2) and the presence or absence of Trp 107 in CDR3. The number of TRDV1 genes evaluated here that occur within each set is also shown (Table 4) with set 1 being the largest and set 4 being the smallest. While some sets can share the same features reported in Table 3 (i.e. sets 5 and 8, 4 and 6) the classification of TRDV1 genes into those separate sets is supported based on the phylogenetic analysis (Figure 4) and additional sequence features (data not shown).

**Table 3:** Features of bovine TRDV1 genes

Phylogenetic set	**Relationship to previously named ovine sets **[15]^1^	CDR1 length	CDR2 (amino acid at IMGT 57)	CDR3 (presence of W at IMGT 107)	Exceptions
1	1C	7	G	-	
2	1B	7	Y	+/-	TRDV1t (CDR1 length of 8)
3	1D/1C	9	E	+/-	
4	1A	9	Y	-	
5	1A	9	Y	+	TRDV1d (Q at IMGT-107)
6	1A	9	Y or N	-	
7	NA	5 or 10	A or V	+	
8	1A	9	Y	+	
9	1E	7	lacks CDR2	+/-	TRDV1o (CDR1 length of 18)
10	1E	7	N or G	+	
11	1E	7	G or V	+	

^1^NA indicates a bovine set lacking a related ovine set.

**Table 4:** TRDV phylogenetic sets

TRDV gene	TRDV1 set^1^	Bovine genes in sets^2^	CDR-IMGT lengths	Number of TRDV genes in each set^2^
TRDV1	1	1a, 1ac, 1af, 1b, 1h, 1k, 1l, 1m, 1n, 1s	[7.3.4]	10
	2	1am, 1an 1az 1t	[7.3.4] [7.3.3] [8.3.4]	4
	3	1aa, 1ah, 1ax, 1bm, 1bn, 1g, 1r	[9.3.4]	7
	4	1c, 1aq	[9.3.4]	2
	5	1aj, 1ap, 1ba, 1bd, 1d	[9.3.4]	5
	6	1ag, 1aw, 1q 1bg 1bj	[9.3.4] [9.3.15] [9.3.12]	5
	7	1bb 1bc, 1ao	[10.3.4] [5.3.4]	3
	8	1ad, 1p, 1x	[9.3.4]	3
	9	1ae, 1ar, 1f 1o	[7.0.4] [18.0.4]	4
	10	1i, 1al, 1u, 1z	[7.3.4]	4
	11	1w, 1j, 1bh, 1v, 1as	[7.3.4]	5
TRDV2	NA	2-1, 2-2	[7.3.5]	2
TRDV3	NA	3-1	[7.3.5]	1
TRDV4	NA	4	[7.5.4]	1

^1^NA indicates TRDV subgroup for which no set is defined.

^2^For TRDV1 subgroup genes or subgroups (for the others).

**Figure 5:**
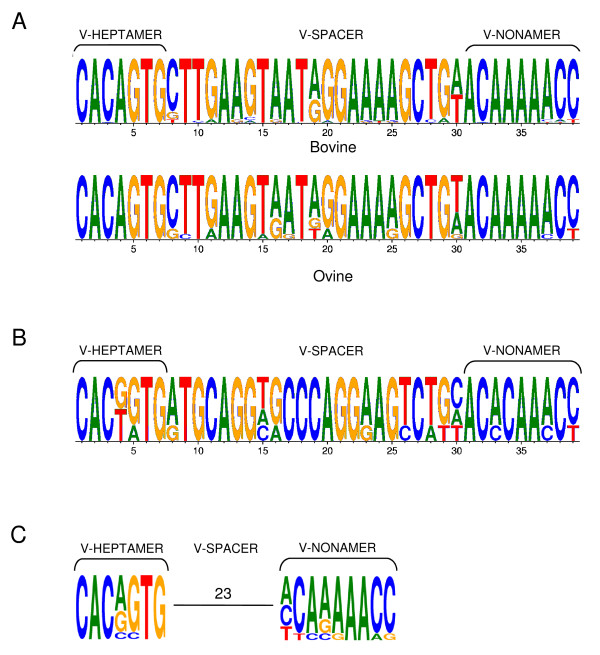
**Comparison of TRDV gene recombination signal sequences**. Sequence logos for recombination signal (RS) sequences are shown for (A) bovine and ovine TRDV1 genes (52 and 12 sequences, respectively). (B) bovine TRDV4 and its ovine, human and murine orthologs (TRDV4, TRDV3 and TRDV5, respectively; four sequences) and (C) remaining human, murine and bovine TRDV genes (human TRDV1, TRDV2; murine TRDV1, TRDV2-1, TRDV2-2, TRDV4; bovine TRDV2-1, TRDV2-2, TRDV3-1; nine sequences). Heptamer and nonamer sequences and spacer lengths are indicated. Logos were generated using WebLogo3 [45].

When bovine TRDV1 RS (total of 52 sequences) and ovine TRDV1 RS (total of 12 sequences) were evaluated, a high level of sequence conservation was observed both among and within species, as depicted in the sequence logos shown in Figure 5A. When the RS of bovine TRDV4 orthologs were compared (including those for human TRDV3, mouse TRDV5 and ovine TRDV4) a similarly high level of sequence conservation was observed among species (Figure 5B). In contrast, comparison of the remaining TRDV RS (those for bovine TRDV2 and TRDV3, ovine TRDV2 and TRDV3, mouse TRDV1, TRDV2 and TRDV4, human TRDV1 and TRDV2) revealed conservation in only the heptamer and nonamer sequences (Figure 5C). This is consistent with the lack of relatedness of these genes as determined based on sequence analysis (refer to Figure 4). Overall, sequence conservation was observed within the RS heptamer and nonamer of all TRDV genes, regardless of species (compare Figure 5A, Figure 5B and Figure 5C).

### TRDD and TRDJ gene analysis

**Figure 6:**
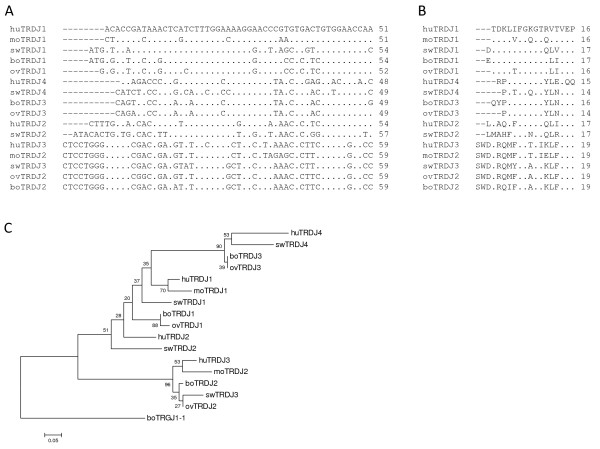
**TRDJ gene sequence alignments and phylogenetic tree**. Alignments of TRDJ gene (A) nucleotide and (B) deduced amino acid sequences, using bovine (bo) genomic TRDJ sequences identified here and previously classified ovine (ov), swine (sw), human (hu) and murine (mo) TRDJ sequences (described in Methods) are shown with identities indicated by a dot (.) and gaps indicated by a dash (-). The Neighbor-Joining method [47] was used to infer evolutionary history of the above mentioned sequences. The optimal tree (C) with the sum of branch length = 2.43145392 is shown. The percentage of replicate trees in which the associated taxa clustered together in the bootstrap test (1000 replicates) are shown next to the branches [48]. The Maximum Composite Likelihood method [49] was used to compute evolutionary distances. Complete deletion to eliminate gaps was performed and the final dataset included a total of 42 positions.

Genomic locations, sequences and RS of bovine TRDD and TRDJ genes were also determined (Table 1 and Additional File 1) and confirmed what had been reported previously [20]. Evidence for five TRDD genes was found and deduced amino acid sequences for all three open reading frames are shown in Additional File  1. Additional putative TRDD genes were identified based on potential RS (data not shown); however, subsequent cDNA sequence analyses provided little or no evidence that those putative TRDD genes were valid and thus they are not presented. Three TRDJ genes were found and their nucleotide and deduced amino acid sequences are also reported in Additional File 1. Alignments comparing bovine, ovine, swine, human and mouse TRDJ gene nucleotide and deduced amino acid sequences are shown (Figure 6A and Figure 6B). A phylogenetic tree (Figure 6C), constructed using the neighbor-joining method to evaluate the relatedness of the above sequences, reflected the alignment.

Bovine, human and murine 5'D-RS and 3'D-RS of TRDD genes and J-RS of TRDJ genes were evaluated for sequence conservation (data not shown). Despite a low number of sequences used in the comparisons (because few TRDD and TRDJ genes exist in the genomes of those species) there appeared to be conserved heptamer and nonamer sequences among the RS for all species evaluated. Although heptamers were found to vary, as expected, they all contained the 5' CAC (or 3' GTG for 5'D-RS and J'RS) consensus sequence which has been found to be critical for recombination [25]. Nonamers generally contained the previously reported consensus sequence ACAAAAACC [34] (or GGTTTTTGT for 5'D-RS and J'RS) although this was found to be less conserved. This is consistent with findings that the nonamer sequence requirements are less rigid than those for the heptamer sequence in order to obtain efficient recombination [25].

### cDNA analysis as evidence for TRD gene usage

Prior to these studies the number of TRDV1 genes found in the bovine genome, and their sequences, had not been resolved. Therefore, it was impossible to classify cloned TRDV1 sequences based on their encoding genes. Furthermore, TRDV1 genes are highly polymorphic but the subgroup also contains genes that differ by only one or three nucleotides in their coding regions (for example TRDV1aa and TRDV1bn, TRDV1v and TRDV1as). Therefore, it has been very difficult to differentiate between expressed TRDV1 sequences with regard to whether they represent the same or different genes. Here we classified previously-identified TRDV1 cloned cDNA sequences based on several criteria which included their relative placement in trees based on nucleotide and deduced amino acid sequences, specific sequence characteristics and percent identities based on nucleotide sequence alignments (data not shown). Results are summarized in Table 5 with the provisional IMGT nomenclature, corresponding genes and TRDV1 sets (as defined in Figure 4 and Tables 3 and 4) indicated.

**Table 5:** Classification of bovine TRDV1 cDNA sequences

cDNA clone name	Accession number^1^	Provisional IMGT nomenclature^2^	Closest germline gene found in this study^3^	Number of nucleotide differences^4^	TRDV set
Vdelta1.36	EF175175	NN	TRDV1l	5	1
BVd1.26	U73393	TRDV1S11	TRDV1m	6	1
SB-C11	FJ907530	NN	TRDV1af	1	1
SB-C17	-	NN	TRDV1af	1	1
BTDV27	D16114	TRDV1S14	TRDV1af	4	1
BVd1.13	U73380	TRDV1S15	TRDV1a	2	1
BTDV2	D13656	TRDV1S18	TRDV1a	2	1
SB-B1	FJ907521	NN	TRDV1a	5	1
SB-C25	FJ907532	NN	TRDV1a	5	1
SB-C3	-	NN	TRDV1a	4	1
BTDV4	D13660	TRDV1S13	TRDV1h	1	1
SB-A5	FJ907519	NN	TRDV1h	6	1
SB-C1	FJ907526	NN	TRDV1h	1	1
SB-C15	-	NN	TRDV1h	1	1
SB-A11	FJ907520	NN	TRDV1h	2	1
SB-C23	-	NN	TRDV1h	1	1
SB-B7	FJ907524	NN	TRDV1b	5	1
BVd1.23	U73390	TRDV1S4	TRDV1b	0	1
SB-C7	FJ907528	NN	ND	NA	1
BTDV1	D16116	TRDV1S6	ND	NA	1
BTDV1	D13655	TRDV1S15	ND	NA	1
BVd1.14	U73381	TRDV1S7	ND	NA	1
SB-B6	FJ907523	NN	ND	NA	1
BTDV8	D16112	TRDV1S12	ND	NA	1
SB-A7	-	NN	ND	NA	1
SB-B11	FJ907525	NN	ND	NA	1
BTDV33	D13658	TRDV1S1	ND	NA	1
SB-C19	-	NN	ND	NA	1
BTDV3	D13657	TRDV1S16	ND	NA	1
SB-B3	FJ907522	NN	ND	NA	1
BTDV28	D16115	TRDV1S17	ND	NA	1
BVd1.24	U73391	TRDV1S2	ND	NA	1
SB-C9	FJ907529	NN	TRDV1an	2	2
BVd1.16	U73383	TRDV1S9	TRDV1an	1	2
BVd1.18	U73385	TRDV1S10	TRDV1an	5	2
BVd1.15	U73382	TRDV1S3	TRDV1am	0	2
SB-C5	FJ907527	NN	TRDV1az^5^	4	2
SB-C13	-	NN	TRDV1az^5^	4	2
SB-A3	-	NN	TRDV1az	1	2
BVd1.17	U73384	TRDV1S8	TRDV1az	1	2
SB-A9	-	NN	TRDV1az	2	2
SB-B9	-	NN	TRDV1az^5^	4	2
BVd1.21	U73388	TRDV1S23	TRDV1ax	1	3
BVd1.20	U73387	TRDV1S22	TRDV1aa	2	3
BTDV6	D13661	TRDV1S24	TRDV1r	1	3
Vdelta1.37	EF175176	NN	TRDV1aq	4^6^	4
Vdelta1.33	EF175171	NN	ND	NA	5
BTDV26	D16113	TRDV1S25	ND	NA	5
BVd1.22	U73389	TRDV1S21	ND	NA	5
BVd1.25	U73392	TRDV1S5	ND	NA	7
Vdelta1.34	EF175172	NN	ND	NA	7
BVd1.19	U73386	TRDV1S20	TRDV1ad	1	8
Vdelta1.35	EF175173	NN	TRDV1ae	0	9
SB-C21	FJ907531	NN	TRDV1u	5	10
BTDV36	D13659	TRDV1S19	ND	NA	11

^1^A dash indicates redundant sequence for which a GenBank accession number was not obtained.

^2^NN indicates sequence for which no Provisional Nomenclature exists.

^3^ND indicates cDNA sequences for which no corresponding gene could be identified in this study.

^4^Number of nucleotides that are different between the Provisional IMGT Nomenclature and the corresponding gene sequences between amino acid positions 1 and 104 (2nd-CYS); NA indicates sequence that lacks corresponding gene.

^5^Genes listed as corresponding are the closest matches; however, classification of these genes was unclear.

^6^cDNA sequence used for comparison was partial.

In some cases it was impossible to classify TRDV1 cDNA sequences definitively, because some TRDV1 genes share very high sequence identity; those cases are indicated in Table 5. Furthermore, in 20 cases (see Table 5) no corresponding TRDV1 genes could be identified although cDNA sequences were found to have features characteristic of particular sets, as indicated. It is possible that no corresponding genes were found because additional TRDV1 genes are present in the bovine genome but were not identified in this study due to gaps in the analyzed regions of the Btau_3.1 assembly. Only cDNA sequences that differed from potential corresponding gene sequences by six or fewer nucleotides were classified; however, it remains possible that those cDNA sequences are not in fact representative of the genes indicated. Overall, corresponding genes were identified for most cDNA sequences (35 out of 55 sequences), although 36 of the 52 predicted TRDV1 genes reported here lacked cDNA confirmation and those genes were found to be distributed among all sets. Last, for the sake of completeness we note that cDNA evidence for bovine TRDV2, TRDV3 and TRDV4 genes reported here has already been demonstrated [17] and has been confirmed [20].

**Figure 7:**
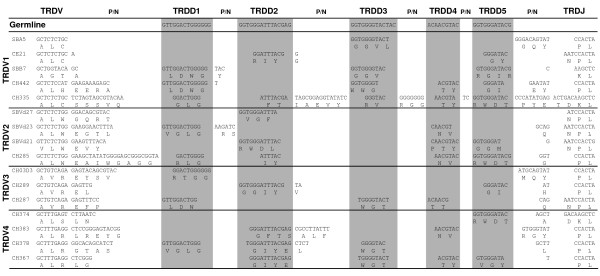
**TRD rearranged CDR3 sequences and TRDD gene usage**. TRD rearranged CDR3 nucleotide sequences, and their corresponding deduced amino acid sequences, derived from cDNA from peripheral blood mononuclear cells, were aligned to demonstrate TRDD usage. Germline TRDD sequences are shown above representative TRDV1, TRDV2, TRDV3 and TRDV4 sequences containing between one and five TRDD genes. TRDD genes are shaded and gene usage was determined by the presence of at least six nucleotides of a particular TRDD gene. Sufficient cDNA sequence was analyzed to determine TRDV gene usage; however, the sequences shown here have been truncated after the 2nd-CYS 104 and at the beginning of the TRDJ gene.

Identification of the TRDD genes within the bovine genome allowed us to evaluate TRDD gene usage in cDNA sequences that had been previously sequenced in our laboratory. CDR3 of 73 TRDV1, 17 TRDV2, 17 TRDV3 and 36 TRDV4 containing sequences were analyzed (Table 6). All D genes were represented in transcripts containing genes of the four TRDV subgroups. P nucleotides were observed flanking untrimmed TRDD regions and extensive N-regions were frequently found between TRDD regions. Some cases were ambiguous because nucleotides could be attributed to either P- or N-region but also to the presence of more than one TRDD gene. Therefore, we applied the criteria that at least six nucleotides of a particular TRDD gene must be present to claim usage of that gene with reasonable confidence. Examples of TRDD gene usage with nucleotide and deduced amino acid sequences are shown (Figure 7). These demonstrated the incorporation of one to five TRDD genes within a single CDR3. Only cases in which the order of TRDD genes in the CDR3 corresponded to that found in the genome were retained.

**Table 6:** TRDD gene usage in CDR3 of bovine rearranged sequences by TRDV genes of different subgroups

TRDV subgroup	Number of sequences evaluated	Number of times TRDD gene identified (Percentage of all TRDD genes used)				
		
		TRDD1	TRDD2	TRDD3	TRDD4	TRDD5
TRDV1	73	33 (24.3)	33 (24.3)	18 (13.2)	23 (16.9)	29 (21.3)
TRDV2	17	6 (16.2)	9 (24.3)	7 (18.9)	9 (24.3)	6 (16.2)
TRDV3	17	9 (29.0)	3 (9.7)	10 (32.2)	4 (12.9)	5 (16.1)
TRDV4	36	9 (14.8)	16 (26.2)	11 (18.0)	16 (26.2)	9 (14.8)

## Discussion

In order to evaluate and characterize the bovine T cell receptor delta gene repertoire and their genomic organization we annotated the TRD genes in the Btau_3.1 assembly. Here we describe the existence of two TRDV2 genes, as well as a single gene each for TRDV3 and TRDV4. We confirmed the presence of three TRDJ and five TRDD genes [20] and evaluated TRD junctional diversity incorporating these genes. Furthermore, we described the existence of 52 TRDV1 genes (46 of them functional and six of them ORF), evaluated them for their sequence characteristics and further classified them into eleven sets based on phylogenetic analysis. As for other mammals, the bovine TRD locus was found embedded within the TRA locus and thus TRDV1 genes were distributed amongst the TRAV genes. Indeed, there were a number of genes for which assignment to the TRAV or TRDV group was ambiguous. It is important to note that the presence of TRAV/DV genes is certain and has been reported [17] but the ability of TRDV or TRAV genes (as defined based on sequence characteristics) to rearrange with either TRDD or TRAJ must be determined experimentally and will be evaluated in future studies. Evaluation of the genes contained within the TRA/TRD locus was reported [22] following the initial submission of this work with overall findings similar to those discussed here.

The question remains as to whether or not the findings reported here represent the complete TRD gene repertoire because of the gaps in the Btau_3.1 assembly. The presence of additional TRDV1 genes is almost certain because of extensive gaps in this region although it is unlikely that additional TRDD or TRDJ genes are present in the genome since that region is fully scaffolded and has been carefully evaluated for the presence of additional genes. That region also contains TRDV4 and its analysis is likely definitive. In addition, previous cDNA evidence supports the existence of only two TRDV2 genes [17] and is consistent with our findings. The existence of two TRDV3 genes has been reported [20] but is not supported by our findings which may be due to gaps in the genome assembly. Even with gaps, locus organization can be assisted by comparison to other species. Indeed, all mammals studied have genes that are closely related to human TRDV1 with some being distributed among the TRAV genes. The bovine TRDV4 gene is located downstream of the single TRDC gene in inverse orientation, as found for the human TRDV3 and mouse TRDV5 genes. It is notable that there is significant sequence conservation among the orthologous bovine TRDV4, human TRDV3, mouse TRDV5 and ovine TRDV4 genes as well as their entire RS. This suggests that they derive from a common ancestral gene that appeared before the separation of these species. By comparison, there are no human or mouse genes that are equivalent to bovine TRDV2 or TRDV3. The enormous expansion of the TRDV1 repertoire that has been observed for cattle, sheep and pig [15,21] is striking when compared with the single TRDV1 gene found in human (and its two orthologs in mouse) and thus does not assist us in predicting the extent of the possible missing information.

TRDV1 gene subgroup expansion is also striking when compared with other TRDV gene subgroups which, even in artiodactyls, are found to contain only one or two members. While the number of TRGV genes found in each species does not vary considerably [31,35,36], expansion of the TRDV1 genes in bovine, sheep and pig is consistent with them being "γδ T cell high" species, implying that this increase in antigen receptor diversity permits them to be stimulated by and to respond to more types of antigens. The presence of fewer TRDV genes in "γδ T cell low" species, such as humans and mice, suggests that these species evolved so that γδ T cells play a less important role in their immune function. Sequence conservation among bovine TRDV1 RS (including the 23-bp spacer sequences), supports the concept that the large TRDV1 subgroup arose from multiple duplication events. Many bovine TRDV1 genes share high percent identities, indicating that in some cases this subgroup is not as diverse as the number of individual genes might suggest. For example, some TRDV1 sequences differed by as few as four (TRDV1m and TRDV1s), three (TRDV1v and TRDV1as) or even one (TRDV1aa and TRDV1bn) nucleotide in their coding regions even though all were determined to be distinct genes based on analysis of the flanking genomic sequences. However, it cannot be ruled out that some of these gene models represent the same gene but do not appear as such due to sequencing and/or assembly anomalies. The question of why numerous and highly similar TRDV1 genes have been maintained in the bovine genome is compelling and studies to evaluate their functions will be pursued.

Based on phylogenetic sequence analysis and gene features, we subdivided the bovine TRDV1 genes into eleven sets. Each set corresponds to intraspecies gene duplications of ancestral genes that were present before the separation between bovine and ovine species. We also assigned bovine cDNA sequences to germline genes identified in this work (see Table 5). Of 55 previously identified TRDV1 cDNA sequences evaluated, 20 could not be assigned to a germline gene, possibly because of gaps in that region of the genome assembly. Of the 52 TRDV1 genes identified in this study, cDNA evidence existed for only 16. It is possible that, because a majority of the previously identified TRDV1 cDNA sequences were derived from peripheral blood mononuclear cells [16-20], the remaining TRDV1 genes that lack cDNA verification might be expressed in other tissues that have not been evaluated. It is also possible that other factors, such as age or immunological experience, might impact the expression of particular genes and thus prevented verification of those genes. Future studies will focus on specifically amplifying individual TRDV1 transcripts in order to verify that they are functional and to determine whether they are expressed in an age and/or tissue dependent manner.

The potential importance of the role of the T cell receptor delta rearranged CDR3 is evident instudies evaluating the binding of the T10 and T22 antigens [26,37,38] by murine γδ T cells since antigen recognition by the CDR3 was found to be autonomous, that is, not dependent on the TRDV gene associated with it [39]. It could be reasoned that, because the genomes of humans and mice lack the extensive TRDV repertoirethat has been observed in artiodactyls, thiswould be compensated for by diversity of the genes that rearrange to form the CDR3 (i.e. TRDD and TRDJ genes). Moreover, because many bovine TRDV1 amino acid sequences are very similar, structurally it might be expected that their ligand binding regionsincluding CDR3 would also need to be as diverse as that in humans and mice. Humans have three TRDD and four TRDJ genes while mice have two TRDD and two TRDJ genes. With the exception of the human TRDJ genes, these numbers all fall short of the five TRDD and three TRDJ genes identified in cattle. We demonstrated here that in cattle the TRD CDR3 contained combinations that include between one and five TRDD genes and would range in length from nine to 37 amino acids. With the exception of TRDD4, the germline bovine TRDD genes predominantly encode glycine and the hydrophobic residues valine and tryptophan, with the neutral residues threonine and tyrosine being encoded to a lesser extent (see Additional File 1). This is reminiscent of findings for the equine VH CDR3 loops in which a higher proportion of glycine and lower proportion of cysteine content was identified when sequences were compared with those from humans, sheep and pigs [40]. As was suggested for equine VH CDR3, this indicates that bovine TRDV CDR3 loops have increased flexibility and are therefore better suited to recognize a large number of antigenic conformations. Considering this, it is unlikely that CDR3 diversity in mice and humans compensates for their less diverse TRDV repertoire and, in fact, it seems that cattle have more scope for antigen binding on all levels.

If the highly diverse CDR3 is indeed the main structural component involved in antigen recognition, then the TRDV gene products might be most important for interactions with co-receptors or antigen presenting cells, whatever their nature may be. While the interactions between γδ T cell receptor and the antigens that they bind are still largely unknown, it has been demonstrated that for αβ T cell receptor the CDR1 loops (which are germline encoded) are involved with binding peptide as well as MHC. The CDR2 loops (also germline encoded) bind only MHC. The TRDV CDR1 in cattle exhibited diverse lengths and were generallyfound to be five to ten amino acids long as for mouse and human TRDV, TRBV and TRAV CDR1. Thisdiverse bovine TRDV CDR1 repertoire may contribute to antigen binding in contrast to what has been reported for murine γδ T cell recognition of T22 as described above. It was found that bovine TRDV1 CDR2 were either only three amino acids long or absent completely (e.g., TRDV1f, TRDV1ae, TRDV1ar and TRDV1o) in contrast to TRBV CDR2 lengths of five to seven amino acids in mammals. It is yet to be determined whether the TRDV1 genes lacking CDR2 are functional, however, if they are found to be so this lackof, or abbreviation of, the CDR2 inbovine TRDV1 genes is logical since γδ T cells are not MHC-restricted.

## Conclusions

Based on annotations of the bovine genome we identified the TRD genes including 56 TRDV genes, five TRDD genes, three TRDJ genes and the single TRDC gene and described their organization within the TRD locus. Furthermore, we report that the TRDV1 subgroup contains 52 genes, indicating that this subgroup underwent expansion as has been found for sheep and swine. Also, because this large gene subgroup has been maintained in the bovine genome this indicates a unique role for these genes in γδ T cell biology.

## Methods

### Genome Annotation

In conjunction with the Bovine Genome Sequencing Consortium http://genomes.arc.georgetown.edu/bovine/, manual annotation of the T cell receptor delta genes was performed using the Apollo Genome Annotation and Curation Tool, version 1.6.5 [41] and the bovine genome assembly Btau_3.1 [42]. First, a BLAST search of bovine T cell receptor delta cDNA sequences against the Bovine Official Gene Set (GLEAN) was performed in order to identify predicted gene models. These were then analyzed using the Apollo software and the following actions were performed where applicable: (i) models were checked for correct exon-intron structure, (ii) initiation and termination codons were identified where applicable and in other cases 5' and 3' ends were set based on RS and splice sites, (iii) exons were either added or deleted if it was determined that the coding region in the predicted model was incorrect, (iv) predicted gene models were split when a single model encompassed more than one gene or merged when two models coded for a single gene and (v) RS were identified for TRDV, TRDD and TRDJ genes. Predicted gene models identified from the BLAST search were considered pseudogenes, and were not included in subsequent analyses, when premature stop codons or frameshifts occurred in areas where the sequence integrity was deemed adequate. Furthermore, predicted gene models were considered Open Reading Frame (ORF), as defined by IMGT [31] if the coding region had an open reading frame but the sequence structure differed significantly from known TRDV sequences; ORF gene models were included in subsequent analyses. Following annotation, predicted gene model identity and classification were verified using BLAST searches and IMGT/V-QUEST [32]; only gene models that were unambiguously identified as TRDV genes, and not TRAV/DV genes, based on those results were included in subsequent analyses.

### Sequence analyses

Nucleotide sequences were aligned and analyzed using BioEdit version 7.0.5.3 [43]. Exon-intron structure schematics were based on alignments of cDNA and genomic DNA sequence using SIM4 [44] and visualized with LalnView http://pbil.univ-lyon1.fr/software/lalnview.html. GLEAN numbers of the annotated bovine gene sequences used in sequence analyses are reported in Table 1. Published T cell receptor delta sequences, derived from cloned RT-PCR products, were submitted to GenBank (http://www.ncbi.nlm.nih.gov/Genbank/index.html; see Table 5 for accession numbers) and assigned to TRDV genes identified here. CDR3 sequence data described in Table 6, from bovine TRD rearranged genes, are available upon request. Rearranged CDR3 lengths were determined from positions 105 to 117, that is, between the 2nd-CYS 104 and J-PHE 118 of the FGXG motif, according to the IMGT unique numbering for V-DOMAIN [29]. The accession numbers of additional sequences used in analyses are as follows: human (*Homo sapiens*): TRDV1 (M22198), TRDV2 (X15207), TRDV3 (M23326), TRDJ1 (M20289, AE000661), TRDJ2 (L36386, AE000661), TRDJ3 (M21508, AE000661), TRDJ4 (AJ249814, AE000661), TRDD1 (M23325), TRDD2 (M22153), TRDD3 (M22152); mouse (*Mus musculus*): TRDV1 (M94080), TRDV2-1 (AE008686), TRDV2-2 (AE008686), TRDV4 (AE008686), TRDV5 (AE008686), TRDJ1 (AF019412), TRDJ2 (X64903, NT_039614), TRDD1 (X64900), TRDD2 (X64901); pig (*Sus scrofa*): TRDJ1 (D49560, AB053451), TRDJ2 (D49561, AB053451), TRDJ3 (D49562, AB053451), TRDJ4 (D49563, AB053451); cattle (*Bos taurus*): TRAV (BC148926), TRGJ1-1; sheep (*Ovis aries*): TRDV1S1 (Z12970, AJ786827), TRDV1S2 (Z12981), TRDV1S3 (Z12988), TRDV1S4 (Z12989), TRDV1S5 (Z12990, AJ786828), TRDV1S7 (Z12992), TRDV1S8 (Z12993, AJ786830), TRDV1S9 (Z12994, AJ786831), TRDV1S10 (Z12971, AJ786832), TRDV1S11 (Z12972), TRDV1S12 (Z12973), TRDV1S13 (Z12974, AJ786833), TRDV1S14 (Z12975), TRDV1S15 (Z12976), TRDV1S16 (AJ786834), TRDV1S17 (Z12978), TRDV1S18 (Z12979, AJ786835), TRDV1S19 (Z12980), TRDV1S20 (Z12982), TRDV1S21 (Z12983), TRDV1S22 (Z12984), TRDV1S24 (Z12986), TRDV1S25 (Z12987), TRDV1S29 (AJ786836), TRDV1S30 (AJ786837), TRDV1S31 (AJ786838), TRDV1S32 (AJ786839), TRDV1S33 (AJ786840), TRDV1S34 (AJ786841), TRDV1S35 (AJ809502), TRDV1S36 (AJ809503), TRDV1S37 (AJ809504), TRDV1S38 (AJ809505), TRDV1S39 (AJ809506), TRDV1S40 (AJ809507), TRDV2 (Z12995), TRDV3 (Z12996), TRDV4 (Z12997, AJ810117), TRDJ1 (AJ277510), TRDJ2 (AJ277511), TRDJ3 (AJ277512).

Multiple sequence alignments were performed using ClustalW2 (http://www.ebi.ac.uk/Tools/clustalw2/index.html; [28]) and the default parameters and were visualized using JalView [30]. IMGT unique numbering was determined for the V-DOMAIN [29] and TRDV secondary structures were evaluated and displayed as IMGT Collier de Perles [33] as determined using IMGT/V-QUEST [32]. Sequence logos were generated using WebLogo3 [45].

### Phylogenetic analyses

Phylogenetic analyses were performed using nucleotide sequences, unless otherwise indicated, of functional (and ORF) TRDV and TRDJ genes. TRDV sequences were truncated to include the V-REGION (IMGT amino acid positions 1 through 108) and comprised those from bovine (identified here in Btau_3.1), human, mouse and ovine (see accession numbers above). TRDJ sequences were truncated to include sequence between the RS (5' end) and splice site (3' end) and comprised those from bovine (identified here in Btau_3.1), human, mouse, ovine and swine (see accession numbers above).

Phylogenies were constructed in MEGA4 [46] using the neighbor-joining method and the p-distance model for nucleotide sequences. Phylogenies were tested using an interior branch test or bootstrap analysis, as indicated, with 1000 replications.

### cDNA analysis

Bovine TRDV cDNA was obtained as described in previous studies [16,17]. Briefly, blood was collected via jugular venipuncture into heparin and peripheral blood mononuclear cells were isolated via density gradient centrifugation over Ficoll-Paque™ PLUS (GE Healthcare Bio-Sciences, Piscataway, NJ) according to the manufacturer's protocol. Animal use complied with federal guidelines and had IACUC approval. Pelleted cells were resuspended in TRIzol (Invitrogen, Carlsbad, CA) and RNA was isolated according to the manufacturer's protocol. Polymerase chain reaction (PCR) was performed following reverse transcription (RT) and using primers designed to specifically amplify TRDV transcripts as previously described [16,17]. In addition, amplification of TRD rearranged CDR3 was performed using a common reverse primer called TRDC-rev (5'-CTC CTT CAC CAA ACA AGC GAC G-3') and the following forward primers: TRDV1S1S2-for (5'-GCA GAT AAA TCC ATC AGC CTC ACC-3'), TRDV1S3-for (5'-CAC AGA ACT CCA TCA GCC TCA CC-3'), TRDV1S4-for (5'-TCA CGT AAA GCC ATC AGC CTC ATT-3'), TRDV2-for (5'-GGA AAA AAA ATC ATC AGC CTC ACC-3'), TRDV3-for (5'-TAC AAA CCC AAC CAA ATG CTG AAA-3'), TRDV4-for (5'-AGC ATG AGC CAA AAA ACC TTC CAC-3'). PCR products were analyzed on TAE agarose gels and cloned into the pCR2.1 vector (Invitrogen) according to the manufacturer's protocol. cDNA clones were sequenced commercially (GeneWiz, South Plainfield, NJ) in order to verify the insert identity and for subsequent sequence analysis.

## Authors' contributions

CH carried out the annotations, molecular studies and the sequence analyses and helped to draft the manuscript. MPL participated in interpretation of data and helped to draft the manuscript. CB participated in the design of the study, interpretation of data, securing funding and helped to draft the manuscript. All authors read and approved the final manuscript.

## Supplementary Material

Additional file 1 Germline TRDD and TRDJ gene sequences Click here for file
