# Supplementary material for: Annotation and classification of the bovine T cell receptor delta genes
Source: BMC Genomics. 2010 Feb 9;11:100. doi: 10.1186/1471-2164-11-100 (PMC2846910; doi:10.1186/1471-2164-11-100)
Supplement: Additional file 1 — Germline TRDD and TRDJ gene sequences [file 1471-2164-11-100-S1.PDF]

**Supplementary Table - Germline TRDD and TRDJ gene sequences**

| Gene | 5’ Nonamer | 5’ Spacer | 5’ Heptamer | TRDD gene coding sequence | 3’ Heptamer | 3’ Spacer | 3’ Nonamer |
|---|---|---|---|---|---|---|---|
| TRDD1 | GGTTTTTGT | AAAGCTCTGTAG | CACTGTG | GTT GGA CTG GGG GG<br>V G L G<br>L D W G<br>W T G G | CACAGTA | TTACAAACCTCAAAGAGACCTCT | ACAGAAACT |
| TRDD2 | GGTTTTTGT | AAAGCTCTGCAG | CACTGTG | GGT GGG ATT TAC GAG<br>G G I Y E<br>V G F T<br>W D L R | CACATAG | CTACAATACCCAAAGAGACCTGT | ACAGAAATT |
| TRDD3 | GGTTTTTGT | AAAGCTCTGTGG | TACTGTG | GGT GGG GTA CTA C<br>G G V L<br>V G Y Y<br>W G T | CACCGCA | ATACAAACATCACACAGACCTGT | ACAGGAACT |
| TRDD4 | GGTTATTGT | AAAGCTGTGCTT | CACTGTG | ACA ACG TAC<br>T T Y<br>Q R<br>N V | CACGGAG | GTTGAAGTGAATTAAATCCTTGT | TCAAAAACC |
| TRDD5 | GGTTTTTGT | AAAGGACTGTAG | CATTGTG | GGT GGG ATA CG<br>G G I<br>V G Y<br>W D T | CACGGTG | ATACAAAACCCACAGAGACCTGT | ACAAAAACT |

| | 5’ Nonamer | 5’ Spacer | 5’ Heptamer | TRDJ gene coding sequence |
|---|---|---|---|---|
| TRDJ1 | GGTTTTTGG | AAAGCCCTCAAG | CTCTGTG | AT GAG ACT GAC AAG CTC ATC TTT GGA AAA GGG ACC CGC CTC ATC GTG GAA CCA A<br>E T D K L I F G K G T R L I V E P |
| TRDJ2 | GTTACTTGT | CAGGTAGTGTCG | CAATGTG | C TCC TGG GAC ACC CGA CAG ATA TTT TTT GGA GCT GGC ACC AAA CTC TTC GTG GAG CCC C<br>S W D T R Q I F F G A G T K L F V E P |
| TRDJ3 | AGTTTTTAG | ACTGGGTTTATC | AGCTGTG | CAG TAT CCA CTA ATA TTT GGC AAA GGA ACC TAT CTG AAC GTG GAA CCA G<br>Q Y P L I F G K G T Y L N V E P |
